# The counterfeit anti-malarial is a crime against humanity: a systematic review of the scientific evidence

**DOI:** 10.1186/1475-2875-13-209

**Published:** 2014-06-02

**Authors:** Kaliyaperumal Karunamoorthi

**Affiliations:** 1 Unit of Medical Entomology and Vector Control, Department of Environmental Health Sciences and Technology, College of Public Health & Medical Sciences, Jimma University, Jimma, Ethiopia

**Keywords:** Malaria, Counterfeit drugs, Counterfeit anti-malarials, Developing economies

## Abstract

**Background:**

The counterfeiting of anti-malarials represents a form of attack on global public health in which fake and substandard anti-malarials serve as *de facto* weapons of mass destruction, particularly in resource-constrained endemic settings, where malaria causes nearly 660,000 preventable deaths and threatens millions of lives annually. It has been estimated that fake anti-malarials contribute to nearly 450,000 preventable deaths every year. This crime against humanity is often underestimated or ignored. This study attempts to describe and characterize the direct and indirect effects of counterfeit anti-malarials on public health, clinical care and socio-economic conditions.

**Methods:**

A search was performed using key databases, WHO documents, and English language search engines. Of 262 potential articles that were identified using a fixed set of criteria, a convenience sample of 105 appropriate articles was selected for this review.

**Results:**

Artemisinin-based combination therapy (ACT) is an important tool in the fight against malaria, but a sizable number of patients are unable to afford to this first-line treatment. Consequently, patients tend to procure cheaper anti-malarials, which may be fake or substandard. Forensic palynology reveals that counterfeits originate in Asia. Fragile drug regulations, ineffective law-enforcement agencies and corruption further burden ailing healthcare facilities. Substandard/fake anti-malarials can cause (a) economic sabotage; (b) therapeutic failure; (c) increased risk of the emergence and spread of resistant strains of *Plasmodium falciparum* and *Plasmodium vivax*; (d) an undermining of trust/confidence in healthcare stakeholders/systems; and, (e) serious side effects or death.

**Conclusion:**

Combating counterfeit anti-malarials is a complex task due to limited resources and poor techniques for the detection and identification of fake anti-malarials. This situation calls for sustainable, global, scientific research and policy change. Further, responsible stakeholders in combination with the synthesis and supply of next generation malaria control tools, such as low-cost anti-malarials, must promote the development of a counterfeit-free and malaria-free future.

## Background

### Counterfeit drugs: a silent tsunami

Counterfeiting is one of the oldest and most profitable occupations [1]. However, even today, it remains difficult to detect, investigate or quantify. The World Health Organization (WHO) estimated that nearly half of the global pharmaceutical market is occupied by counterfeit drugs [2]. Recent estimates suggest that nearly 800 fake drug types [3] are within the legitimate pharmaceutical market structure, and globally there is a massive increase in counterfeit or falsified drug sales to over US$75 billion in 2010, an increase of more than 92% from 2005 [4]. Poverty, weak economies, poor regulatory systems, short supply, and the rising cost of therapeutic agents have created a corresponding increase in production of fake drugs because of the huge profit margin [5].

Both substandard and counterfeit drugs are serious problems, and remain as one of the most neglected public health issues [6], where counterfeiting is mounting to more than 60% in Third World countries [7]. WHO estimates about 60% of purchased counterfeited products did not have any active pharmaceutical ingredient (API), 17% contain too much or too little API, whilst another 16% contain the wrong ingredients altogether [8].

### Types of counterfeiting

The rising threat posed by counterfeited or falsified medicines (see definitions on Table 1) is aggravated by two factors: (i) the most common types of fake drugs, called ‘lifestyle drugs’ are those used to treat erectile dysfunction or baldness; and, (ii) ‘lifesaving drugs’, meant to prevent or treat asthma, malaria, cancer, HIV/AIDS, tuberculosis, blood pressure and heart conditions, diabetes, and severe diarrhoea [9]. From 2007 to 2009, online counterfeit medicine trade rose from US$4 to 11 billion via over 2,930 fraudulent websites [10].

**Table 1:** Key definitions

**Life saving drug**	A pharmaceutical product designed for or used in saving lives.
**Lifestyle drug**	A pharmaceutical product characterized as improving the quality of life rather than alleviating or curing disease.
**Spurious/falsely-labelled/falsified/counterfeit (SFFC) medicines**	Medicines that are deliberately and fraudulently mislabeled with respect to identity and/or source.
**Counterfeit drug**	▪ Deliberately and fraudulently produced and/or mislabeled with respect to identity and/or source to make it appear to be a genuine product.
	▪ It contains less or more than the required amount of active pharmaceutical ingredients (API) used in the authentic version or even contains the correct amount of API but have been manufactured in unsanitary and unsafe conditions [11].
**Substandard drug**	Genuine drug products which do not meet quality specifications set for them. If substandard drugs are knowingly produced to make an unlawful product, they too are considered counterfeit.
**Falsified drug**	It usually has no active ingredient or dangerous substances and can cause serious harm to patients.
**Gray pharmaceutical**	Space is emerging where illicit profiteers are ostensibly marketing competitive brands without regulatory approval.

In general, the industrialized economies suffer with life-style related diseases, whereas in the emerging economies infectious diseases are a matter of grave concern. As a result, both the life saving and lifestyle drugs are much prone to counterfeiting. Besides, people have a tendency to buy cheaper alternatives paving a way for a big market of fake drugs.

**Figure 1:**
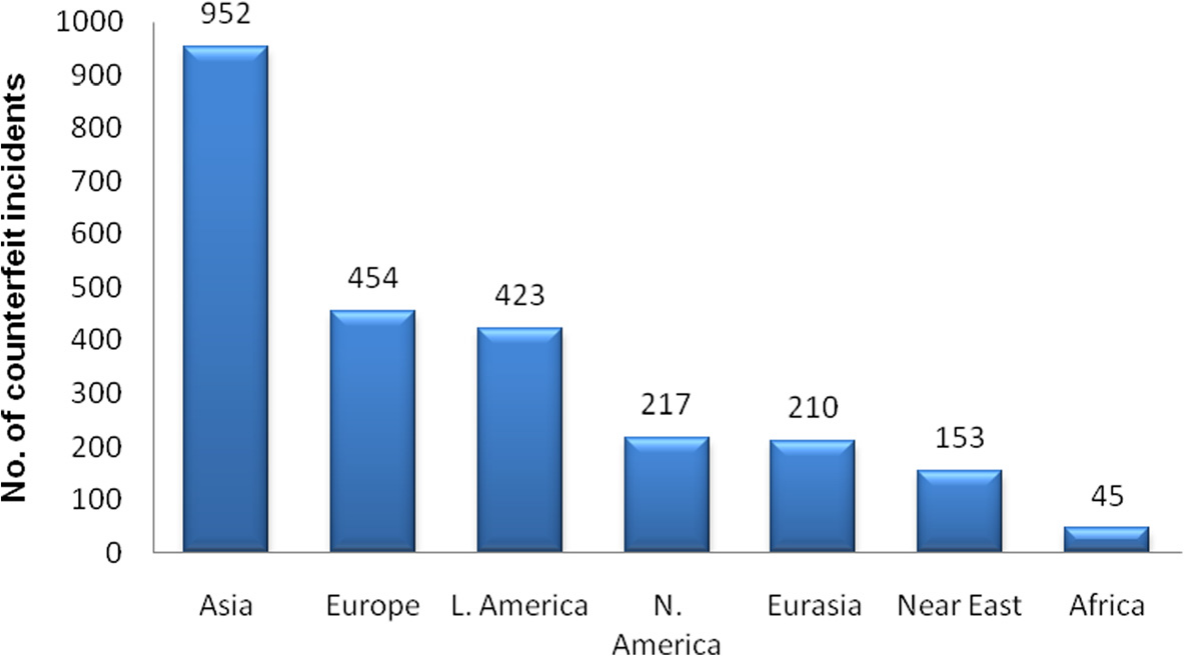
**Pharmaceutical crime incidents by region, 2012 (Source: Pharmaceutical Security Institute, 2013)**[11]**.**

Nevertheless, the magnitude of this problem is not well-known or remains scanty. So far, no study has been conducted to determine and quantify the exact extent of counterfeiting [11]. Figure 1 shows the clear trend of pharmaceutical crime incidents in 2012. Although the crime incident is extremely low in Africa as per the report of pharmaceutical security institute [11], it is the major victim of counterfeit drugs, particularly fake anti-malarials, due to low socio-economic status, fragile drug-regulatory mechanisms and weak law-enforcement agencies. These agencies are often understaffed and underfunded too.

Fake drugs have flourished due to the emergence and resurgence of many infectious diseases, particularly the three major killer diseases: HIV/AIDs, malaria and tuberculosis. It is important to note that these diseases are more common, widespread and destructive in resource-poor settings of Africa and Asia. Although the traffic of counterfeit anti-malarials poses a grave threat to all sections of society, it imposes severe negative socio-economic, clinical and public health concerns, particularly in the endemic settings of sub-Saharan Africa and Southeast Asia. Over decades, the silent ‘tsunami’ of substandard and fake anti-malarials is poorly understood, undervalued or ignored, which has resulted in hundreds of thousands of preventable deaths and considerable morbidity every year. Tragically, it is often tacit, unreported or under-reported in several low- and middle-income countries (LMICs).

There is a vast literature on the impact of counterfeit drugs. However, to the best of the author’s knowledge and understanding, only a limited number of studies report on the negative impact of counterfeit anti-malarials. In this perspective, the present scrutiny becomes more significant and pertinent and is an attempt to determine the global impact of counterfeit anti-malarials in terms of clinical, public health and socio-economic concerns. It also identifies the potential associated-risk factors and delineates emerging opportunities. It could provide some baseline information for policy-makers, public healthcare experts, and other stakeholders to design and implement appropriate strategies to confront this modern-age inhuman act. It may empower not only a counterfeit-free anti-malarial world, but also establish the ambitious goal of a malaria-free world through sustainable, global, scientific research and policies.

## Methods

### Identification of studies

The search strategy and terms were developed collaboratively with an information specialist. An appropriate search was conducted in PubMed, Google Scholar, Web of Science, EMBASE, Scopus, WHO’s WHOLIS, US Pharmacopeia databases, anti-counterfeiting networks, and search engines in English, using the terms: ‘Anti-malarials/analysis’ or ‘Anti-malarials/standards analysis and packaging of anti-malarial drugs in Southeast Asia and sub-Saharan Africa’, ‘counterfeit drugs’, ‘substandard drugs’, ‘falsified drugs’, ‘artemisinins’, ‘artesunate’, ‘drug resistance’, ‘sub-standard anti-malarial’ and ‘counterfeit anti-malarial’ to include articles published without time limitations, i.e., from earliest to most recent (August 2013). Potentially relevant papers (in all languages) were accessed in order to review the full text. The references cited by each relevant paper, review and book chapter were scrutinized to locate additional potential papers. In addition, the reference lists of all articles for which the full text was reviewed were hand-searched, and the full text of those references that appeared relevant to counterfeit drugs and counterfeit anti-malarials were retrieved.

### Inclusion criteria

**Figure 2:**
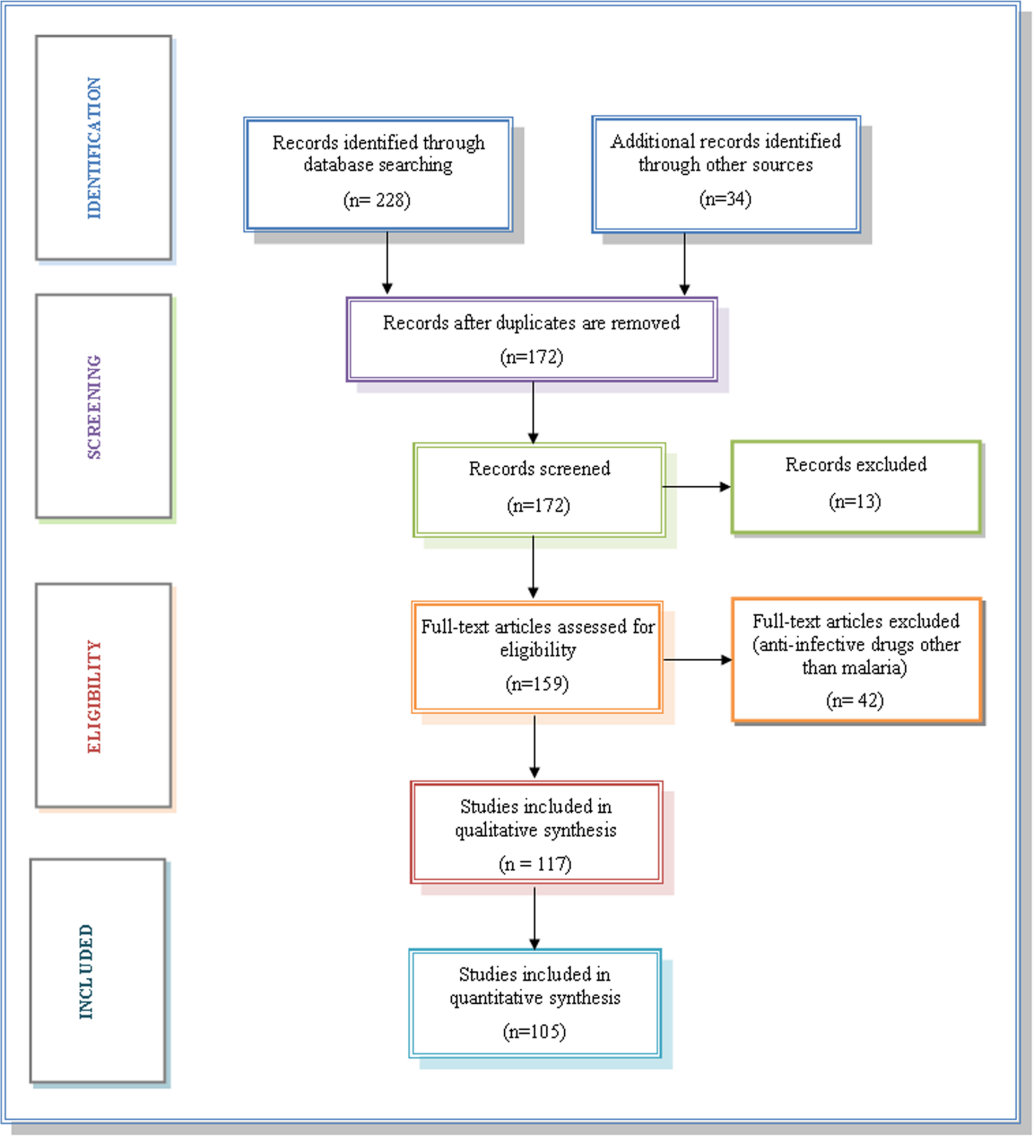
PRISMA flow diagram showing the exclusion and inclusion of studies for analysis.

Studies that reported primary data on the prevalence and potential public health, and clinical and socio-economic concerns of counterfeit anti-malarials, were selected by using the Preferred Reporting Items for Systematic Reviews and Meta-Analyses (PRISMA) guideline procedure. The titles and abstracts of the identified articles are checked against predetermined criteria for eligibility (the studies that deal with the various aspects of counterfeit anti-malarials) and relevant to the study objectives. Where multiple publications presented identical data, the most ‘informative version’ of the study was included. Studies published in a language other than English were translated, and relevant papers were included. The full text of all thus selected studies was obtained, and assessed. Of 262 potential articles identified by these criteria, 117 studies were included for qualitative (dealing with phenomena that are difficult or impossible to quantify mathematically) synthesis and 105 of them were used for the quantitative synthesis (dealing with phenomena that are possible to quantify mathematically) by using the convenience sample method (A statistical method of drawing representative data by selecting appropriate studies because of their ease of access) for obtaining relevant data presented in this systematic review (Figure 2). Articles were retrieved from the important databases and search engines, and were further refined with bibliographic search of these articles.

### Exclusion criteria

In the beginning, a total of 262 (228 + 34) articles were identified through searching various databases (228) and other sources (34). However, 90 articles were rejected because of repetition and the resultant 172 articles were chosen. In the screening phase, 13 studies were excluded as they reported on lifestyle-related drugs, such as diabetes, erectile dysfunction and baldness. Subsequently in the eligibility phase, 42 articles were eliminated as they describe anti-infective counterfeit drugs, except malaria (Figure 2). Nevertheless, it is significant to emphasize that these excluded studies provided an in-depth knowledge for the investigator on the overall prevalence of counterfeit medicines and their negative impact both in emerging economies and developed countries.

### Risk of bias assessment

Two reviewers independently assessed and screened the search results by title and abstract for potential eligibility. Subsequently, the full texts of potentially suitable articles were obtained and were further screened for inclusion. The disagreements in the screening of full texts were resolved by a third reviewer with expertise in fake anti-malarials and this was required for three of the 159 screened papers. Consequently the included studies were further independently assessed for risk of bias. For observational study designs, risk of bias was evaluated using a simple *pro forma* for three domains, namely selection bias, information bias (differential misclassification and non-differential misclassification), and confounding. Risk of bias for each domain was assessed as either low, unclear or high. Studies that had a low risk of bias in each domain, including a low risk of confounding, were classified as having a low overall risk of bias. Qualitative studies were evaluated for quality using an adapted version of a checklist used in a previous series of mixed methods systematic reviews incorporating both quantitative and qualitative studies [12,13].

### Data synthesis and analysis

A data extraction form was developed in Microsoft Excel. The key details of the included studies are presented in the Table, sorted by country, year of publication, major study findings and by first author (see Additional file 1). In addition to the details in the present review, pharmaceutical crime incidents by region, 2012 [11], reports of poor-quality artesunate (ART) in Africa [6] and reports of counterfeit anti-malarials in Southeast Asia [14], are supplemented in terms of graphical presentations. Meta-analysis was not performed as included studies were heterogeneous in important aspects, including populations (different ages and settings), study designs (cross-sectional, case-control, cohort) and variable definitions (including different definitions of counterfeit drugs, particularly anti-malarial and outcomes).

## Results

**Figure 3:**
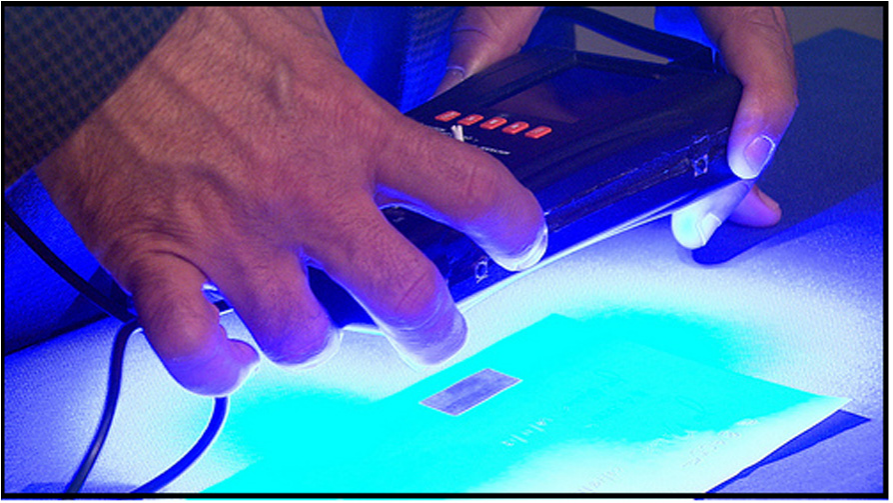
**Counterfeit Detection Device (CD-3) (Credit: US-FDA, 2013)**[60].

The study selection process is shown in Figure 3 as a PRISMA flow diagram. Of 262 potentially relevant, unique citations from all literature searches, 105 studies met the inclusion criteria. Sixty-two studies were empirical research studies, three were book chapters, two were from conference, and 14 involved meta-analyses, with the remaining 18 involving case reports or press releases. Nearly half of the studies were carried out in the WHO-defined African region (n = 50), fifteen in the Southeast Asian region, nine were out of WHO-conducted studies and reports, and the remaining thirty studies from the rest of the world; the majority of them were published after 1990. About nine out of ten studies described the prevalence, existing challenges, emerging opportunities and public health impact of fake or substandard anti-malarials in malaria-endemic settings of Africa and Southeast Asia. The remaining studies examined the actual extent of counterfeit drugs as an issue of clinical, public health and socio-economic concern worldwide.

## Discussion

### Impact of counterfeit drugs: quite avoidable

Counterfeiting undermines public trust, credibility with healthcare systems/providers, and potentially leads to non-adherence of patients to their life-saving drugs or importation of expensive, branded medicines [15]. The quality of commercially available drugs varies greatly among countries, due to the lack of strict regulations and to deficient quality control practices. In some countries, even the amount of active ingredient may be incorrect. In addition, poor formulation techniques can greatly affect the quality of medicine by slow-release of active ingredients. Sometimes drugs may be contaminated with other substances and by poor storage conditions, when humid, tropical environments may contribute to chemical degradation of several pharmaceuticals [16].

### Emerging economies: an opportunity of counterfeiters

Less-developed countries are often a target for counterfeiters, since the cost of legitimate drugs may be unaffordable for a large proportion of a sizable population, and consequently patients prefer to use cheaper versions than expensive, genuine ones. In addition, regulatory and law enforcement systems remain weak. Counterfeiting of lifestyle drugs such as Viagra or cancer medicines is more widespread in developed countries [17]. Counterfeiters are opportunistic criminals motivated by profits yet with limited legal penalties [18], and they produce fake drugs out of starch, chalk and a variety of toxic active ingredients [19,20], for diseases, such as malaria, that severely affect the underprivileged section of society [7].

A study found that the majority of the fake anti-malarials originated from east Asia [6] and distributed through different channels, such as government and private hospitals, pharmacies or other legitimate or illegitimate distributors in the emerging economies [21]. Many counterfeit medications remain undetected within the legitimate supply chain [18]. It has been estimated that fake drugs for malaria and tuberculosis potentially kill over 700,000 people every year [22]. One third of anti-malarial drugs in disease-endemic regions of sub-Saharan Africa are fake [23]. This is an issue that needs to be dealt with via regional, national and international policies [24] with workable cooperation.

### Global burden of malaria

Malaria continues to be a massive global public health problem and it is prevalent in 104 countries. It has been estimated that nearly half of the world population (3.4 billion people) is at risk [25,26]. Although it is a preventable and curable illness, it remains one of the most important causes of maternal and childhood morbidity and mortality in sub-Saharan Africa [27]. WHO estimates that there were about 219 million cases of malaria in 2012 (with an uncertainty range of 154 million to 289 million) with more than 627 000 deaths (Table 2). Most deaths have occurred among children under five years of age living in Africa, where a child dies from malaria for every single minute. Tragically, in Africa, where 80% of malaria cases are treated at home, the disease infact kills one child in 20 before the age of five [26].

**Table 2:** **Malaria an overview (WHO, 2013)**[26]

**Population at risk**	
**3.4 billion**	Nearly half of the world population.
**Clinical cases (2012)**	
**207 million**	Clinical cases.
**80%**	Estimated malaria cases occur in Africa.
**Global deaths (2012)**	
**627 000**	Globally
**90%**	Deaths were from Africa
**80%**	Estimated malaria deaths occur in 18 most affected countries
**77%**	Deaths were among children under 5 years of age
**40%**	Malaria deaths occurs in Nigeria and Democratic Republic of Congo
**Endemic countries (2013)**	
**104**	Endemic countries
**97**	On-going malaria transmission
**06 countries**	Account for 47% of malaria cases, (an estimated 103 million)
	- Nigeria
	- Democratic Republic of the Congo
	- United Republic of Tanzania
	- Uganda
	- Mozambique
	- Cote d’Ivoire
**Economic cost**	
**12 billion US$**	Per year in direct losses, lost; 1.3% of GDP growth per year for Africa.
**Costs of interventions**	
**1.39 US$**	Long-lasting insecticidal net that lasts for three years: per person per year of protection
**0.90 - 1.40 US$**	Course of artemisinin-based combination therapy (ACT) for an adult
**0.30 - 0.40 US$**	Course of artemisinin-based combination therapy (ACT) for an young child
**0.50 US$**	Rapid diagnostic test

### Existing malaria control interventions: good companions

Malaria is a disease of poverty and a cause of poverty [28]. It is quite understandable that countries with higher proportions of their population living in poverty (less than US$1.25 per person per day) have higher mortality rates from malaria [26]. It imposes a severe negative public health and socio-economic impact on Third World countries, where malaria is likely to be endemic (hypo-, meso-, hyper-, or holo-endemic) in nature [28]. At the moment, in many malaria-endemic countries, malaria control is one of the formidable challenges due to: (a) widespread resistance against conventional chemical insecticides, particularly the emergence of ‘behavioural resistance’ to pyrethroid ITNs in 64 countries [29]; (b) lack of sufficient access to reliable diagnostic tools and rational treatment with ACT [30]; (c) widespread poverty and ecosystem degradation [28]; (d) fragile healthcare systems [28]; (e) emergence and spread of multidrug-resistant strains of *P. falciparum*; and, (f) lack of reliable vaccine [30-32].

Despite these challenges, this decade is considered to be significant in malaria history. This is because over the past ten years there has been an impressive increase in international funding and substantial commitment by governments of endemic countries, donors and global malaria partners for the unprecedented scale-up of effective malaria prevention, control and elimination strategies. Consequently, the international community can postulate the long-term, ambitious goal of malaria elimination [32].

Vector control is considered to be a cornerstone of malaria control: to manage vector populations and eventually to reduce/interrupt the disease transmission. Existing malaria control interventions offer highly encouraging results and have afforded a unique opportunity to eliminate malaria in a few countries [32]. The WHO estimates that the malaria related illness and mortality have been dramatically declining [26]. There is a ray of hope as the malaria map shrinks and malaria incidence falls and some countries may consider attacking the remaining foci with an elimination agenda by employing front-line vector control tools such as the distribution of long-lasting, insecticide-treated mosquito nets, effective case management with potent anti-malarials, selective intradomicillary spraying and source reduction [33]. However, the heavy reliance on pyrethroids has led to the emergence of resistance in a wide variety of malaria-endemic settings [34]. It is considered to be a potential threat to the global public health [35]. The continuous persistence of substandard and fake anti-malarials further aggravates the synthesis and spread of resistant strains against affordable drugs such as chloroquine, which ultimately led to an amendment of treatment policy to costlier anti-malarials, such as ACT in malaria-endemic countries [36].

### Artemisinin combination therapy: a hope

#### Mode of mechanism

The use of combination chemotherapy is the current innovative strategy to effectively combat malaria. It involves the use of a short half-life artemisinin drug in combination with a long half-life conventional tablet [37]. The artemisinin kills the parasites and is excreted quickly, resulting in the re-emergence of the parasites after a period of time, but when it is administered in combination with a longer half-life anti-malarial, it achieves complete eradication of the parasites and averts the recrudescence that occurs with the use of artemisinin monotherapy [38].

WHO recommends ACT as first-line treatment for falciparum malaria worldwide [39]. In addition, parenteral artesunate, an artemisinin derivative, became the treatment of choice for severe malaria in adults after it was shown to reduce mortality by 35% compared with quinine [40]. Artemisinin derivatives are characterized by a rapid mode of action against gametocytes (the sexual stages) of the parasite that infects mosquitoes [41,42]. In 2011, 278 million courses of ACT were procured for the public and private sectors in endemic countries - up from 182 million in 2010, and just 11 million in 2005 [26], and in Fiscal Year 2012, the President’s Malaria Initiative procured more than 73 million treatments.

#### A potential target of modern counterfeiters

ACT is one of the potential anti-malarials that has been well-tolerated. It saves millions of lives every year. The artemisinin compounds are derived from the plant Qinghao (*Artemisia annua;* Asteraceae), which takes nearly six months to grow and produces artemisinin. Its chemical modification has led to a series of potential anti-malarial drugs (artemether, ART and arteether) [25]. It is in short supply and there is massive demand [7]. In addition, ACT is relatively expensive: the cost of a course for an adult and a young child is US$ 1.20-3.50 and 0.30-0.40, respectively [43]. There is demand for cheaper versions amongst the poorest and most vulnerable in society, which is the major driving force to establish counterfeit anti-malarial industries to produce and distribute fake anti-malarials in Third World countries, where widespread corruption, insufficient drug regulation and weak law enforcement are common [6,44].

Insufficient amenities and ability to detect and identify the quality of anti-malarials, poor consumer and health-worker knowledge about these drugs and lack of punitive action (inflicting a punishment) against counterfeiters make these drugs attractive targets for counterfeiters [44,45]. Sophisticated counterfeits have been reported in Asia [46] resulting in a number of cases of increased morbidity and mortality in recent years [7] which has generated concern that a similar problem would emerge in Africa [7,47]. Poorer populations living in rural, remote areas and international travellers/tourists from non-endemic areas are paying a high price for the counterfeit anti-malarials. Fake ART has been reported to be circulating in Africa at least since 2001 [7].

#### Counterfeit anti-malarials: a silent epidemic in malaria-endemic settings

Substandard and counterfeit anti-malarial drugs are a foremost concern in developing economies, particularly in the holo/hyper-endemic settings of sub-Saharan Africa [11] and Southeast Asia. Medicines for Malaria Venture (MMV) recognizes that counterfeiting of anti-malarials is a multibillion-dollar business, with well sophisticated operators who evade detection by drug-regulatory authorities [48]. WHO estimates that fake anti-malarials cause up to 20% (200,000 people) of the one million malaria deaths worldwide each year. A recent International Policy Network (IPN) study [11] estimates the actual burden could lead to 450,000 preventable deaths every year.

ART has become the target of an extremely sophisticated and prolific counterfeit drug trade, both in terms of ART tablets and packaging [7,47,49,50]. The counterfeit/substandard anti-malarial medicines may contain inappropriate concentrations of active ingredients, contamination with other drugs or toxic impurities, poor quality ingredients, poor stability and inadequate packaging [51]. The endemic countries of sub-Saharan Africa and Southeast Asia have become more vulnerable to counterfeiting, and are badly affected by fake anti-malarials. They were often failed for chemical analysis, packaging analysis, or were falsified.

### Epidemiology

#### Counterfeit anti-malarials in Africa: required stringent actions

Over the past decade, a dramatic rise in fake anti-malarials has become one of the top public health issues in sub-Saharan Africa. The illegal production, distribution and sale of counterfeit drugs represent more than 50% of the pharmaceutical market in several African countries [52]. Interpol estimates that 30% of anti-malarials circulating in Africa are either counterfeit or of inferior quality. A recent study found that 44% of samples from Senegal and 30% from Madagascar could be qualified as of inferior quality [53]. Seven collections of artemisinin derivative monotherapy, ACT and halofantrine anti-malarial of suspicious quality were collected in 2002/10 in 11 African countries (see Figure 1 in reference [6]) and substandard/counterfeit or degraded ART and ART + amodiaquine were found in eight countries. Pollen analysis reveals that the fake drugs originated from East Asia [6].

#### Counterfeit anti-malarials in Southeast Asia: educate the public

In Asia, counterfeiting of anti-malarials is the major concern in China, Thailand, Indonesia, Vietnam, Cambodia, and Laos [54]. Surveys indicate that up to 40% of ART was counterfeit in this region. Lack of any active ingredient is a typical problem, but there are fakes with the correct ingredients yet with deficient dose, or in fake packaging, or containing the wrong ingredients [54]. Newton *et al.*[14] found that of 104 shop-bought ‘artesunate’ samples from Cambodia, Laos, Myanmar (Burma), Thailand, and Vietnam, 38% did not contain ART.

In an unprecedented, international collaboration, a study found that half of the samples [(49.9% (195/391)] contained no active ingredient or too little to have any benefit. Chemical analysis demonstrated a wide diversity of the wrong active ingredients, including banned pharmaceuticals and carcinogens [55]. Dondorp *et al.*[49] reported that tablet packs are labelled as ‘artesunate’ but 53% did not contain any active ingredient. Fake blister packs are often hard to distinguish from their genuine counterparts. Of the 44 mefloquine samples, 9% contained <10% of the expected amount of active ingredient. ACT were found to be counterfeit in 38 and 53% in two studies conducted in different countries in Southeast Asia [7,16]. In Cambodia it has been shown that fake ART was sold by 71% of local drug vendors [56]. Although several studies have reported on fake and substandard anti-malarials, just a few important studies are listed in Additional file 1.

### Impact on public health

#### Impact of counterfeit anti-malarials: preventable

Fake anti-malarials usually contain no or less active ingredient so that the unwitting patient is at substantial risk of developing severe malaria and dying [49]. Seiter [15] tried to quantify the cost of ineffective anti-malarials from prior estimates; he predicted that in a country of about 20 million people, there may be four million treatments and nearly 800,000 cases treated with poor quality anti-malarials causing up to 4,000 childhood deaths, and which also contributes to the genesis of resistance because of subtherapeutic dosage. Besides, it favours the selection and spread of resistant strains [44] (Table 3), making it tougher to treat malaria, even with authentic medicines. Therefore, poor-quality anti-malarials are very likely to jeopardize the progress and investment in control and elimination of malaria made in the past decade [23].

**Table 3:** Negative impacts of counterfeit antimalarials

**S. No.**	**Negative Socio-cultural-clinical-Public Health Impacts**
**1.**	Economic sabotage
**2.**	Therapeutic failure
**3.**	Increase the risk of emergence and spread of resistance strains
**4.**	Undervalue *the trust/confidence* on *healthcare* stakeholders
**5.**	Loss of trust on healthcare systems
**6.**	Adverse side-effects
**7.**	Even death

Evidently, the recent emergence of artemisinin resistance or tolerance of the *P. falciparum* strain in the Thailand-Cambodia border [57] makes protection of effective drug supply imperative [23]. As fake anti-malarials cannot be easily distinguished from genuine, the confidence of the public healthcare providers and systems at large is undermined [49]. Substandard and counterfeit anti-malarials are directly or indirectly associated with higher morbidity, mortality and stunted socio-economic growth due to additional healthcare costs because of treatment failure, and impose a severe burden of disability adjusted life years (DALYs) at national level [52].

### Factors promoting counterfeiting of anti-malarials

#### Need better understanding: assessing the truths and managing the key issues

Although the counterfeit anti-malarial trade is a global phenomenon, it is extremely difficult to assess the actual extent worldwide, particularly in low-and middle-income countries where malaria is a major public health issue and there is a higher prevalence of fake drugs, mainly due to: (a) limited financial resources; (b) paucity of skilled manpower; (c) absence or weak anti-counterfeiting laws; and, (d) poor judicial entities, and corruption. The problem is varied between countries and even between regions within the same country, although industrialized countries do have stringent regulatory mechanisms to ensure the quality of drugs.

#### Inadequate healthcare facilities

Many endemic countries have a free ACT treatment policy. However, the rural poor, particularly those living in remote areas have to walk/travel a long distance to access free treatment. Many villages are inaccessible by road, and only a limited number of health facilities have a regular supply of ACT [25]. Eventually, these people will deal with drug vendors, who knowingly or unknowingly sell fake or substandard drugs, which has ultimately led to the emergence of drug resistance and treatment-failure, and severe morbidity and mortality.

#### Lack of awareness: unwitting

In endemic regions anti-malarials are widely distributed and self-prescribed (incorrectly and correctly) for many febrile episodes attributed to malaria [23]. Fake drugs are highly sophisticated, including convincing packaging, holograms and marketing, and represent a major technical and law enforcement challenge [55]. In general, poor patients and public healthcare stakeholders have inadequate awareness on fake anti-malarials and their potential hazards [58]. Licensed distributors, pharmacists, health care providers, or patients are unable to distinguish authentic medicines [21], which mean the issue is prone to under-reporting biases. A study of the packaging of the genuine and counterfeit anti-malarials sold in Uganda [59] shows that it is extremely difficult, even for educated personnel, to distinguish them.

### Emerging opportunities: looking to the future -new science and policies

Fraud of anti-malarials is well-organized and widespread in Southeast Asia and Africa; it can be confronted by multilateral, global, scientific research and multinational approaches that implement multiple, parallel, anti-counterfeit strategies with a strong political commitment by the regional, national and international networks and cooperation protocols (Table 4). Therefore, identifying and adopting existing as well as emerging opportunities to preserve the efficiency of one of the key arsenals, such as ACT, is paramount in the fight against malaria, otherwise fake anti-malarials may lead to serious, global, clinical, public health disaster in the near future.

**Table 4:** Emerging opportunities to eliminate fake antimalarials/malaria

**S. No.**	**Suggested interventions**
**1.**	Community-based anti-counterfeit campaigns
**2.**	Determine the actual extend and nature of the problem
**3.**	Strict law enforcement agency against all the perpetrators
**4.**	Setting up quality drug surveillance systems
**5.**	Developing affordable next generation antimalarials
**6.**	Removal of taxes and tariffs on antimalarials and other commodities of malaria control
**7.**	Sharing the knowledge and logistical information within and between the endemic countries
**8.**	Launching and maintaining the regional, national and international databases

#### Needs more precise prevalence: real insight required

The counterfeit industry is dominating nearly half of the pharmaceutical industry and has become a billion-dollar business. However, acquiring reliable estimates on the clinical, public health and socio-economic impact is imprecise and unclear [53]. Consequently, today’s estimates or predictions may likely be the tip of the iceberg. Assessing the actual nature, extent and cost of the counterfeit issue will improve our understanding, and inform priorities in terms of resource mobilization and allocation to address this crisis appropriately. It may facilitate the identification and implementation of appropriate anti‒counterfeit strategies by bolstering the evaluation of existing interventions and the implementation of cost‒effective practices [53].

#### Innovative next generation tools and simple techniques: achievable

Counterfeiters are well funded with sophisticated equipment to manufacture and supply fake anti-malarials. Currently a number of different approaches are being undertaken to identify fake drugs. However, lack of inexpensive tools is one of the major impediments for their elimination/eradication. Recently, the US Food and Drug Administration (FDA) [60] developed a hand-held counterfeit detection device (CD-3) (Figure 3) to identify counterfeit or substandard anti-malarials, including falsified products. CD-3 illuminates a product with a variety of wavelengths of light to provide a visual comparison of an unverified product with an authentic sample (Figure 3). This allows inspectors to identify counterfeit anti-malarials and remove them from the supply chain. Minimal scientific or technical background is enough to operate the device and it can be used even in remote communities or in places with only very basic health care systems [60]. In fact, it is a low-cost and effective counterfeit drug-testing tool that was already in usage in 50 FDA field laboratories. A study is under-way to determine the effectiveness of the CD-3 in Ghana [60] and it will be available in all malaria endemic settings very soon.

Besides sample chemical profiling, researchers use forensic palynology to study pollen contamination within fake drugs in order to detect the probable location of manufacture enabling the fight against fake medicines [61]. However, only a limited number of laboratories are equipped to perform thorough forensic chemistry of fakes.

Further scientific research and development is called for to design user-friendly, low-cost and robust portable (hand-held) devices and techniques for drug quality screening and assessment, and for the detecting and identifying of marker compounds and chemical profiling [62] to determine authenticity of anti-malarials in resource-constrained regions of Asia and Africa in real settings.

### Counter-measures

#### Global policy on counterfeit drugs: calls for immediate stringent measures

Initially fake antimalarials have received a very less attention than the other malaria control interventions. However, significant efforts have been made, and more stringent measures are needed to curb this harmful practice effectively [52]. Public health institutions, ministries and attorneys must take the lead in countering poor-quality drugs. A balance has to be struck between the need to make affordable anti-malarials available to everyone and ensure the quality of drugs without compromise [63]. Furthermore, establishing an international counterfeit database is imperative to obtain a strategic and operational intelligence in the fight against fakes [64]. Additionally, there is a need for a global anti-counterfeit strategy to regulate and monitor pharmaceutical industries and drug markets, with provision at international, national, as well as regional-base for the regulation of all medicines, particularly anti-malarials, including alternative and complementary medicines by adopting local conditions and demands. These can ensure access to quality anti-malarials in the future.

#### Require strict law-enforcing agency: strict with the principles

Countries do try their best, but the counterfeiting industry is well organised and does not even shy away from killing government officials. For instance Dr. Dora Akunyili, the former Director General of the National Agency for Food and Drug Administration and Control (NAFDAC), Nigeria had been targeted by a drug gang of counterfeit medicine, since she had put them out of business. Finally, she has expressed as follows ‘malaria can be prevented, HIV/AIDS can be avoided and armed robbery may kill a few at a time, but fake drugs kill en masse [65]’.

Therefore, speedy criminal investigations and legal actions against all perpetrators, including manufacturers and distributors [49], is imperative to dismantle or disrupt the illicit trade of fake anti-malarials. Counterfeiting of medicines is a crime against intellectual property with civil and/or administrative penalties under specific acts, rather than as a public health threat warranting criminal sanctions [66]. The weakness of federal drug regulation agencies and systems is one of the key elements favouring the spread and persistence of counterfeit drugs. Therefore, stringent pre- and post- anti-malarial drug surveillance is vital in all malaria-endemic countries of sub-Saharan Africa and Southeast Asia [36], with strong law enforcement and adequate monetary support and resources. Tragically, these core elements are deficient in most malaria-endemic settings.

It has been estimated that out of the 193 WHO member states, only about 20% are known to have well-developed drug policies and law enforcement agencies. Only 50% of the member states implement regulations at various levels and 30% have no medicinal regulation in place [67]. However, active collaboration at all levels (regional, national and international) is a key to annihilate fake medicine by sharing the logistic information and technical skills with cross-border approaches. These key demands must be fulfilled; otherwise, fake anti-malarials may likely to jeopardize the impressive gains of the past decade in malaria control.

#### Development of affordable novel anti-malarials: unavoidable

In past decades significant efforts have been made to develop new anti-malarials to evade the emergence and spread of resistance, by means of new bio-active molecules with novel mechanisms. The persistent threat calls for continual action [104]. Recent genome-based technologies and *in vitro* screening of whole parasites have broadened the range of therapeutic targets and have accelerated the development of new generation anti-malarials [68]. MMV is committed to building a strong pipeline of molecules leading to the next generation of high-quality anti-malarials to attack malaria parasites through different mechanisms [48].

Over the centuries, more than a thousand plant species have been used as anti-malarial agents in resource-poor settings. However, their clinical efficacy, quality, and safety are a matter of grave concern. Plant-derived compounds will still be an essential aspect of the therapeutic array of new anti-malarials [25]. As the world’s potential anti-malarials, such as quinine and artemisinin, have been derived from the ancient herbal remedy for malaria, it has driven several researchers to develop and deploy an affordable anti-malarial to attack the various stages of malarial parasites. The cheaper anti-malarials can be formulated by forming a vibrant anti-malarial discovery pipeline by bringing all stakeholders, such as traditional healers, ethnobotanists, scientists, medical entomologists, pharmacists, universities, and research institutions to a common platform to isolate and identify lead compounds to develop the next generation of affordable anti-malarials to combat multidrug-resistant strains effectively [25].

#### Free supply of potent anti-malarials: ambition

It has been estimated that one-third and nearly half of the anti-malarials are fake or substandard in resource-constrained settings of sub-Saharan Africa and Southeast Asia, respectively. It is well known that malaria is a disease of poverty and a cause of poverty [28] and the majority of victims belong to low socio-economic status and are unable to access more expensive ACT. Consequently, they procure cheaper fake anti-malarials. An appropriate drug distribution policy, ideally low-priced or free genuine drugs [52], would hamper the counterfeit industry. It is indeed a Herculean task, but one that can be attained with the pecuniary aid of international/national donors, malaria control partners, and non-governmental organizations. In addition, even if anti-malarials are produced at very low cost or donated for free, it can become expensive along the supply chain to the end user. Therefore, adequate measures must be identified and implemented to confront these barriers.

#### Health education campaigns and programmes

Health professionals should be self-motivated and actively involved in educating the public and patients about counterfeiting in terms of identification, prevention and the repercussions. Pharmacists, nurses and physicians, as well as pharmaceutical companies and their distributors need to be remaining vigilant about counterfeiting in order to best prepare for encounters with suspicious products [24]. All countries, interested parties and stakeholders must act together on a common platform to improve the quality and supply of several life-saving drugs, particularly anti-malarials.

### Study limitations and strengths

When considering the findings of this review, the risk of publication bias cannot be ruled out, particularly for positive findings relating to counterfeit anti-malarials, where it is possible for the studies with null findings to be under-published. It was not possible to produce an Egger funnel plot to formally assess the risk of publication bias, for the same reasons that meta-analysis was not performed, namely the heterogeneity in the study designs, outcome measures, analysis strategies, and populations in the included studies. Reporting bias within individual studies may also be a factor, many of which might have explored the effects of multiple factors on fake anti-malarials. The incomplete retrieval of identifying research and the lack of published protocols for the included studies did not allow the author to estimate the magnitude of this potential bias. The strength of the review is the addition of forward and backward searching methods to the initial database search for articles. A number of additional studies were identified using these methods, before reaching a saturation point at which the only relevant studies identified were included.

## Conclusion

Counterfeiting of anti-malarials is an attack on global public health. This persistent threat has torn society by imposing severe menace in all spheres of clinical, socio-economic, and public health. The majority of victims often belong to underprivileged sections of society. They primarily prefer self-medication from informal, private, pharmaceutical sectors owing to the inaccessibility and unaffordability of quality healthcare medicines. The counterfeiters produce fake anti-malarials and distribute in resource-poor settings. Fortunately, there is an unprecedented awareness among all stakeholders due to adverse repercussions of fake anti-malarials. An urgent and intensified effort is imperative in terms of multi-layered approaches and multi-disciplinary scientific research and policies to prevent a public health disaster.

In order to combat global counterfeiting, the findings of this scrutiny emphasize the following stringent measures to ensure the availability of lower-priced, genuine anti-malarials: (a) determining the actual extent of the problem; (b) community-based, anti-counterfeit campaigns; (c) strict law enforcement agencies against all perpetrators; (d) setting up quality drug surveillance systems; (e) developing affordable next generation anti-malarials; (f) removal of taxes and tariffs on anti-malarials and other commodities of malaria control; (g) sharing the knowledge and logistical information within and between the endemic countries; (h) launching and maintaining national and international databases; (i) establishing adequate analytical laboratories; (j) imposition of price controls; (k) post-market surveillance; (l) supply chain security for active pharmaceutical ingredients and medicinal products; and (m) distribution of low-cost (ideally free) potent anti-malarial through health care facilities and community healthcare workers. These integrated and multivalent strategies could save millions of avoidable malaria-related illnesses and deaths, and ensure access to potent anti-malarial tools to prevent the emergence/spread of multidrug-resistant strains. This shall offer the opportunity to build not only counterfeit-free world, but also the long-term goal of a malaria-free future.

## Competing interests

The author has declared there are no competing interests.

## Supplementary Material

Additional file 1 Summary of the key studies on counterfeit/substandard/falsified anti-malarials in the malaria-endemic settings. Click here for file

## References

[B1] Wertheimer AI Wang PG Counterfeit Medicines Policy, Economics, and Counter Measures 2012 1 St Albans: ILM Publications

[B2] WHO Counterfeit Medicines. Fact Sheet 2006 275 http://www.who.int/mediacentre/factsheets/fs275/en/. Accessed on 15^th^ June 2013

[B3] Redpath S Trade in illegal medicine hits pharmaceutical sector, in World Finance 20 April 2012, World News Media 2012 Online

[B4] Pitts P Counterfeit drug sales to reach $75 billion by 2010, report say Heartland Institute November 2005. http://www.heartland.org/publications/health%20care/article/17948/Counterfeit_Drug_Sales_to_Reach_75_Billion_by_2010_Report_Says.html. Accessed on 06^th^ May 2013

[B5] WHPA Background Document on Counterfeit Medicines in Asia WHPA Regional Workshop on Counterfeit Medical Products, Taipei, Taiwan 30th June– 1st July 2011 http://www.whpa.org/Background_document_Counterfeit_Medicines_in_Asia.pdf. Accessed on 12^th^ June 2014

[B6] Newton PN Green MD Mildenhall DC Plançon A Nettey H Nyadong L Hostetler DM Swamidoss I Harris GA Powell K Timmermans AE Amin AA Opuni SK Barbereau S Faurant C Soong RC Faure K Thevanayagam J Fernandes P Kaur H Angus B Stepniewska K Guerin PJ Fernández FM Poor quality vital anti-malarials in Africa-an urgent neglected public health priority Malar J 2011 10 352 10.1186/1475-2875-10-352 22152094 PMC3262771

[B7] Newton PN McGready R Fernandez F Green MD Sunjio M Bruneton C Phanouvong S Millet P Whitty CJ Talisuna AO Proux S Christophel EM Malenga G Singhasivanon P Bojang K Kaur H Palmer K Day NP Greenwood BM Nosten F White NJ Manslaughter by fake artesunate in Asia-will Africa be next? PLoS Med 2006 3 e197 10.1371/journal.pmed.0030197 16752952 PMC1475657

[B8] WHO The quality of Antimalarials: A Study in Seven African Countries 2003 Geneva, Switzerland: World Health Organization Available at http://whqlibdoc.who.int/hq/2003/WHO_EDM_PAR_2003.4.pdf. Accessed on 10th June 2013

[B9] Lybecker KM Rx roulette: Counterfeit pharmaceuticals in developing nations 2003 Online document available at: http://idei.fr/doc/conf/pha/lybecker.pdf. Accessed on 12^th^ June 2014

[B10] Felman F MarkMonitor finds online drug brand abuse is growing 2009 https://www.markmonitor.com/pressreleases/2009/pr090928-bji.php

[B11] PSI Pharmaceutical Crime incidents by Region 2012 Online document available at: http://www.psi-inc.org/geographicDistributions.cfm. Accessed on 10th June 2013

[B12] Rees R Harden A Brunton G Oliver S Oakley A Young People and Physical Activity: A Systematic Review of Barriers and Facilitators 2001 London: EPPI-Centre, Social Science Research Unit, Institute of Education, University of London

[B13] Harden A Rees R Shepherd J Brunton G Oliver S Oakley A Young People and Mental Health: A Systematic Review of Research on Barriers and Facilitators 2001 London: EPPI-Centre, Social Science Research Unit

[B14] Newton P Proux S Green M Smithuis F Rozendaal J Prakongpan S Chotivanich K Mayxay M Looareesuwan S Farrar J Nosten F White NJ Fake artesunate in Southeast Asia Lancet 2001 357 1948 1950 10.1016/S0140-6736(00)05085-6 11425421

[B15] Seiter A Health and economic consequences of counterfeit drugs Clin Pharmacol Ther 2009 85 576 578 doi:10.1038/clpt.2009.47 10.1038/clpt.2009.47 19451909

[B16] Hall KA Newton PN Green MD De Veij M Vandenabeele P Pizzanelli D Mayxay M Dondorp A Fernandez FM Characterization of counterfeit artesunate antimalarial tablets from southeast Asia Am J Trop Med Hyg 2006 75 804 811 17123969

[B17] Editorial Counterfeit drugs: a growing global threat Lancet 2012 379 685 doi:10.1016/S0140-6736(12)60289-X 10.1016/S0140-6736(12)60289-X 22364747

[B18] Chaudhry PE Stumpf SA The challenge of curbing counterfeit prescription drug growth: Preventing the perfect storm Bus Horiz 2013 56 189 197 10.1016/j.bushor.2012.11.003

[B19] Fernández M Cody RB Green MD Hampton CY McGready R Sengaloundeth S White NJ Newton PN Characterization of solid counterfeit drug samples by desorption electrospray ionization and direct-analysis-in-real-time coupled to time-of-flight mass spectrometry Chem Med Chem 2006 1 702 705 10.1002/cmdc.200600041 16902921

[B20] Alter K Fernandez F Green M Newton PN Analysis of counterfeit antimalarial drugs Eur Pharm Rev 2004 3 1 5

[B21] WHO Report of the situation of counterfeit medicines based on data collection tool WHO Regions for Africa and Eastern Mediterranean. WHO/ACM/3 2010

[B22] International Policy Network (IPN) Fake drugs kill over 700,000 people every year - new report 2009 http://archive.today/ipW8i. Accessed on 12^th^ June 2014

[B23] Nayyar GML Breman JG Newton PN Herrington J Poor-quality antimalarial drugs in SouthEast Asia and sub-Saharan Africa Lancet Infect Dis 2012 12 488 496 10.1016/S1473-3099(12)70064-6 22632187

[B24] Nsimba SE Problems associated with substandard and counterfeit drugs in developing countries: a review article on global implications of counterfeit drugs in the era of antiretroviral (ARVs) drugs in a free market economy East Afr J Pub Health 2008 5 205 210 19374325 10.4314/eajph.v5i3.39004

[B25] Karunamoorthi K Sabesan S Jegajeevanram K Vijayalakshmi J The role of traditional anti-malarial plants in the battle against global malaria burden Vector Borne Zoonotic Dis 2013 13 521 544 doi:10.1089/vbz.2011.0946 10.1089/vbz.2011.0946 23930972

[B26] WHO: World Malaria Report The World Health Organization 2013 Geneva: Switzerland

[B27] Karunamoorthi K Deboch B Tafere Y Knowledge and practice concerning malaria, insecticide-treated net (ITN) utilization and antimalarial treatment among pregnant women attending specialist antenatal clinics J Pub Health 2010 18 559 566 10.1007/s10389-010-0335-9

[B28] Karunamoorthi K Global Malaria Burden: Socialomics Implications J Socialomics 2012 1 e108 doi:10.4172/jsc.1000e108

[B29] Karunamoorthi K Medicinal and aromatic plants: a major source of green pesticides/risk-reduced pesticides J Med Aromat Plants 2013 1 e137 doi:10.4172/2167-0412.1000e137

[B30] Karunamoorthi K Malaria vaccine: a future hope to curtail the global malaria burden Int J Prev Med 2014 5 529 538 24932383 PMC4050672

[B31] Karunamoorthi K Sabesan S Relative efficacy of repellents treated wristbands against three major mosquitoes (Diptera: Culicidae) vectors of disease, under laboratory conditions Int Health 2009 1 173 177 10.1016/j.inhe.2009.08.005 24036563

[B32] Karunamoorthi K Vector control: a cornerstone in the malaria elimination campaign Clin Microbiol Infect 2011 17 1608 1616 doi:10.1111/j.1469-0691.2011.03664.x 10.1111/j.1469-0691.2011.03664.x 21996100

[B33] Karunamoorthi K Peterson AM, Calamandrei GE Global Malaria Eradication: Is It Still Achievable and Practicable? Malaria: Etiology, Pathogenesis and Treatments 2012 New York, USA: Nova

[B34] Karunamoorthi K Tsehaye E Ethnomedicinal knowledge, belief and self-reported practice of local inhabitants on traditional antimalarial plants and phytotherapy J Ethnopharmacol 2012 141 143 150 doi:10.1016/j.jep.2012.02.012 10.1016/j.jep.2012.02.012 22366533

[B35] Karunamoorthi K Sabesan S Insecticide resistance in insect vectors of disease with special reference to mosquitoes: a potential threat to global public health Health Scope 2013 2 4 18

[B36] Tipke M Diallo S Coulibaly B Storzinger D Hoppe-Tichy T Sie A Müller O Substandard anti-malarial drugs in Burkina Faso Malar J 2008 7 95 doi:10.1186/1475-2875-7-95. 10.1186/1475-2875-7-95 18505584 PMC2426704

[B37] Nosten F van Vugt M Price R Effects of artesunate-melfloquine combination on incidence of *Plasmodium falciparum* malaria and melfloquine resistance in Western Thailand: A prospective study Lancet 2000 356 297 302 10.1016/S0140-6736(00)02505-8 11071185

[B38] Walsh JJ Coughlan D Heneghan N Gaynor C Bell A A novel artemisinin - quinine hybrid with potent antimalarial activity Bioorg Med Chem Lett 2007 17 3599 3602 10.1016/j.bmcl.2007.04.054 17482816

[B39] WHO Guidelines for the Treatment of Malaria 2010 Second http://www.who.int/malaria/publications/atoz/9789241547925/en/index.html. Accessed 29^th^ March 2012

[B40] Carrara VI Zwang J Ashley EA Price RN Stepniewska K Barends M Brockman A Anderson T McGready R Phaiphun L Proux S van Vugt M Hutagalung R Lwin KM Phyo AP Preechapornkul P Imwong M Pukrittayakamee S Singhasivanon P White NJ Nosten F Changes in the treatment responses to artesunate-mefloquine on the northwestern border of Thailand during 13 years of continuous deployment PLoS One 2009 4 e4551 10.1371/journal.pone.0004551 19234601 PMC2641001

[B41] Navaratman V Mansor SM Sit NW Grace J Li Q Olliaro P Pharmacokinetics of artemisinin-type compounds Clin Pharmacokinet 2000 39 254 270 10.2165/00003088-200039040-00002 11069212

[B42] Ridley RG Medical need, scientific opportunity and the drive for antimalarial drugs Nature 2002 415 686 693 10.1038/415686a 11832957

[B43] Breman JG Alilio MS Mills A The Intolerable Burden of Malaria II: What's New, What's Needed Supplement to Volume 71(2) of the American Journal of Tropical Medicine and Hygiene 2004 American Society of Tropical Medicine and Hygiene: Northbrook, IL

[B44] Newton PN Green MD Fernández FM Day NPJ White NJ Counterfeit anti-infective drugs Lancet Infect Dis 2006 6 602 613 10.1016/S1473-3099(06)70581-3 16931411

[B45] Bate R Making a Killing; The Deadly Implications of the Counterfeit Drug Trade 2008 Washington DC: USA: The American Enterprise Institute Press

[B46] Fernandez FM Green MD Newton PN Prevalence and detection of counterfeit pharmaceuticals: a mini review Ind Eng Chem Res 2007 47 585 590

[B47] Newton PN Dondorp A Green M Mayxay M White NJ Counterfeit artesunate antimalarials in Southeast Asia Lancet 2003 362 169 12867121 10.1016/S0140-6736(03)13872-X PMC7135335

[B48] Reddy D Banerji J Counterfeit antimalarial drugs Lancet 2012 12 829 23099079 10.1016/S1473-3099(12)70237-2

[B49] Dondorp AM Newton PN Mayxay M Van Damme W Smithuis FM Yeung S Petit A Lynam AJ Johnson A Hien TT McGready R Farrar JJ Looareesuwan S Day NP Green MD White NJ Fake antimalarials in Southeast Asia are a major impediment to malaria control: multinational cross-sectional survey on the prevalence of fake antimalarials Trop Med Int Health 2004 12 1241 1246 15598255 10.1111/j.1365-3156.2004.01342.x

[B50] Aldhous P Murder by medicine Nature 2005 434 132 136 10.1038/434132a 15758966

[B51] Caudron JM Ford N Henkens M Macé C Kidle-Monroe R Pinel J Substandard medicines in resource-poor settings: A problem that can no longer be ignored Trop Med Int Health 2008 13 1062 1072 10.1111/j.1365-3156.2008.02106.x 18631318

[B52] Ambroise-Thomas P P The tragedy caused by fake antimalarial drugs Mediterr J Hematol Infect Dis 2012 4 e2012027 doi:10.4084/MJHID.2012.027 22708042 10.4084/MJHID.2012.027 PMC3375661

[B53] Wilson J Fenoff R The Health and Economic Effects of Counterfeit Pharmaceuticals in Africa Global Edge Business Rev 2011 5 6 1 2

[B54] Parry J WHO combats counterfeit malaria drugs in Asia BMJ 2005 330 1044 doi:10.1136/bmj.330.7499.1044-d 15879383 10.1136/bmj.330.7499.1044-d PMC557259

[B55] Newton PN Fernández FM Plançon A Mildenhall DC Green MD Ziyong L Christophel EM Phanouvong S Howells S McIntosh E Laurin P Blum N Hampton CY Faure K Nyadong L Soong CW Santoso B Zhiguang W Newton J Palmer A Collaborative epidemiological investigation into the criminal fake artesunate trade in south East Asia PLoS Med 2008 5 e32 doi:10.1371/journal.pmed.0050032 10.1371/journal.pmed.0050032 18271620 PMC2235893

[B56] Rozendaal J Fake antimalaria drugs in Cambodia Lancet 2001 357 890 11265991 10.1016/s0140-6736(05)71830-4

[B57] Dondorp AM Yeung S White L Nguon C Day NP Socheat D von Seidlein L Artemisinin resistance: current status and scenarios for containment Nat Rev Microbiol 2010 8 272 280 20208550 10.1038/nrmicro2331

[B58] Moride Y Haramburu F Requejo AA Begaud B Underreporting of adverse drug reactions in general practice Br J Clin Pharmacol 1997 43 177 181 9131950 10.1046/j.1365-2125.1997.05417.x PMC2042725

[B59] McLaughlin KE Counterfeit Medicine from Asia threatens Lives in Africa. The medication on the left is authentic; the right, fakes that may contain little or no active ingredients. Uganda 2012 http://pulitzercenter.org/projects/china-africa-developing-world-corruption-anti-malarial-drugs-health-aid (accessed on 10th June 2013)

[B60] US-FDA FDA launches partnership to protect against counterfeit anti-malarial medicines with FDA-developed handheld detection tool 2013 http://www.fda.gov/NewsEvents/Newsroom/PressAnnouncements/ucm349195.htm. Accessed on 10^th^ June 2013

[B61] Degardin K Roggo Y Been F Margot P Detection and chemical profiling of medicine counterfeits by Raman spectroscopy and chemometrics Anal Chim Acta 2011 705 334 341 10.1016/j.aca.2011.07.043 21962376

[B62] Ricci C Nyading L Yang F Fernandez FM Brown CD Newton PN Kazarian SG Assessment of hand-held Raman instrumentation for in situ screening for potentially counterfeit artesunate antimalarial tablets by FT-Raman spectroscopy and direct ionization mass spectrometry Anal Chim Acta 2008 623 178 186 10.1016/j.aca.2008.06.007 18620922

[B63] Amin AA Kokwaro GO Antimalarial drug quality in Africa J Clin Pharm Ther 2007 32 429 440 doi:10.1111/j.1365-2710.2007.00847.x 10.1111/j.1365-2710.2007.00847.x 17875107 PMC2653781

[B64] The Wellcome Trust Fake Antimalarial Drugs Analysis Highlights Threat to Global Health Press release 12 February 2008. http://www.wellcome.ac.uk/News/Media-office/Press-releases/2008/WTX043187.htm. Accessed on 07th June 2013

[B65] Akunyili D DG NAFDAC[http://naijapositive.myfastforum.org/archive/dr.-dora-akunyili-dg-nafdac__o_t__t_199.html]

[B66] Singh R The Threat of Counterfeit Medicines: a Silent Epidemic Graduate Research and Development in Society, University of Cambridge 2012 http://camtriplehelix.com/journal/issue/17/the-threat-of-counterfeit-medicines-a-silent-epidemic/pdf. Accessed on 09th June 2013

[B67] WHO General Information on Counterfeit Medicines 2013 http://www.who.int/medicines/services/counterfeit/overview/en/index1.html. Accessed on 07th June 2013

[B68] Wells TNC Alonso PL Gutteridge WE New medicines to improve control and contribute to the eradication of malaria Nature 2009 8 879 891 10.1038/nrd2972 19834482

