# Supplementary material for: The counterfeit anti-malarial is a crime against humanity: a systematic review of the scientific evidence
Source: Malar J. 2014 Jun 2;13:209. doi: 10.1186/1475-2875-13-209 (PMC4064812; doi:10.1186/1475-2875-13-209)
Supplement: Additional file 1 — Summary of the key studies on counterfeit/substandard/falsified anti-malarials in the malaria-endemic settings. [file 1475-2875-13-209-S1.doc]

**Additional file 1** Summary of the key studies on counterfeit/substandard/falsified anti-malarials in the malaria-endemic settings

| **Year** | **Study location(s)** | **Anti-malarial(s)** | **Description** | **Reference** |
| --- | --- | --- | --- | --- |
| 1600s | NA | Cinchona bark | The bark has been reported as fake. | Newton *et al.* [10] |
| 1700s | Europe | Cinchona bark | The bark has been reported as fake. | Newton *et al.* [10] |
| 1800s | London | QN | Fake quinine reported | Newton *et al.* [10] |
| 1985 | Kenya | Anti-malarials | The problem of modern-day counterfeit drugs was first addressed at a conference | Ambroise-Thomas, 2012 [6] |
| 1988 | Sierrra Leone | CQ | A cause of Reye's syndrome, has been used in the manufacture of fake CQ in Africa | Sesay [11] |
| 1992 | Nigeria | SP | No active ingredients | ten Ham [12] |
| 1995 | Guinea | CQ | Wrong active ingredient | WHO [13] |
| 1995 | Amazonian region | Primaquine | Excessive as well as reduced active ingredient identified. | Petralanda [14] |
| 1995 | Sudan | CQ | No active ingredient | Abdi *et al.* [15] |
| 1996 | Namibia | Primaquine | Different morphology of fake primaquine tablets is reported | Kron [16] |
| 1997 | Thailand and Nigeria | CQ | 36.5% samples were substandard with respect to pharmacopoeial limits. | Shakoor *et al.* [17] |
| 1998 | Uganda | CQ | Upto 30% tablet samples and 33% injection samples contained less than the stated amount of the active ingredient. | Ogwal-Okeng *et al.* [18] |
| 1998 | Uganda | CQ | Up to 30% tablet samples and 33% injection samples contained less than the stated amount of the active ingredient. | Ogwal-Okeng *et al.* [18] |
| 1999–2000 | Cambodia, Laos, Myanmar, Thailand/Burma border, Vietnam | ART | Demonstrated that 38% ART tablets contained no active ingredient | Newton et al. [2] |
| 2001 | Cambodia, Laos, Myanmar,Thailand, Vietnam | ART | 38% did not contain ART. | Newton *et al*. [2] |
| 2001 | Cambodia | MQ | Fake MQ by 60% | Rozendaal [7] |
| 2001 | Cambodia | ART | Fake ART was sold by 71% | Rozendaal [7] |
| 2001 | Senegal | MQ | It is unlikely that the tablets obtained in Dakar contained the proper amount of MQ | Reidenberg and Conner [19] |
| 2001 | Lao PDR | ART | 38% oral ART collected, by convenience sampling were counterfeit. | Newton *et al*. [2] |
| 2001–2002 | Cambodia, Laos, Myanmar, Thailand/Burma border, Vietnam | ART | 53% artemisinin-based anti-malarials contained incorrect levels of active ingredient. | Dondorp et al. [4] |
| 2002 | Tanzania | CQ | Five formulations (three paracetamol and two chloroquine) failed to meet the dissolution tolerance limits | Risha *et al*. [20] |
| 2002 | Peru | ART | Up to 80% samples of ART contain no active ingredient and are estimated to be fake. | Kapp [21] |
| 2002 | Ghana | Halofantrine (paediatric anti-malarial syrup) | Not publicized by GlaxoSmithKline; the company later admitted that Halofantrine counterfeit medicine was also present in Sierra Leone and Nigeria; had this been shared earlier, the harm could have been reversed. | Wolff et al. [22] |
| 2003 | Gabon, Ghana, Kenya, Mali, Mozambique, Sudan, Zimbabwe | CQandSP | Percentage failures in ingredient content ranging from 20 to 67% for CQ tablet and 5 to 38% for SP tablet and dissolution failures ranging from 5 to 29% for CQ and 75 to 100% for SP. | WHO [1] |
| 2003 | Uganda | CQ tablets and injection dosage forms | 39% failed the content test with 11% having subnormal and 28% having supranormal amounts, whilst 51% injection samples failed with 40 and 11% having subnormal and supranormal amounts, respectively. | Ogwal-Okeng *et al*. [23] |
| 2003 | Tanzania | SP andAQ | Among the AQ samples collected, 13% failed dissolution test, but all passed assay for content, whereas 11 and 44% SP samples failed assay for content and dissolution tests, respectively. | Minzi *et al.* [24] |
| 2004 | Thailand, Lao PDR, Cambodia and Myanmar | MQ | 9% were substandard | Dondorp *et al*. [4] |
| 2004 | Lao PDR | ART | 54% oral ART collected, by convenience sampling were counterfeit. | Dondorp *et al.* [4] |
| 2004 | Cameroon | CQ | 38% CQ, 74% QN, and 12% antifolates had either no active ingredient, an insufficient active ingredient, the wrong ingredient, or unknown ingredient(s). | Basco [25] |
| 2004 | Rwanda and Tanzania | QN and SP | The drug content of all the formulations was within the limits recommended by the USP 24, but after six-month storage, the drug content of one sulphamethoxazole/trimethoprim and one QN formulation were found to be substandard. | Kayumba *et al.* [26] |
| 2004 | Laos | Anti-malarial drugs | 22% sampled anti-malarial drugs had incorrect levels of active ingredient. | Stevens and Mydin [42] |
| 2005 | Cambodia | MQ | 77% quinine sulphate tablets were counterfeit, this is alarming because quinine sulphate is given to severe malaria cases | Smine *et al*. [27] |
| 2005 | Kenya | SP and AQ | Overall, approximately 45.3% SP and 33.0% AQ samples were found to be substandard. | Amin *et al.* [28] |
| 2005 | Cote d’Ivoire | CQ | Very high concentration of active ingredient | Legris [29] |
| 2006 | Laos, Myanmar, Vietnam, Cambodia | ART | 68%ART did not contain correct amount of active ingredient | Hall [5] |
| 2006 | Cambodia | ART, QN, CQ, MQ | 27% anti-malarial drugs tested were found to have incorrect levels of active ingredient. 79% samples were not registered at the Department of Drugs and Food | Lon *et al.* [30] |
| 2006 | Thailand | Several potential anti-malarials | 15.4% ART, 11.1% CQ and 29.4% QN were substandard. | Vijaykadga *et al.* [31] |
| 2007 | Laos, Myanmar, Vietnam, Cambodia | Modern anti-malarials | In some areas 30-50%, or more than 50% drugs bought randomly from pharmacies were fake | McGivering [32] |
| 2007 | **Congo, Burundi and Angola** | QN, CQ, SP andMQ | Only 89% of declared active substance was found in QN tablets, with high quantities of impurities reported. | Gaudiano *et al*. [33] |
| 2007 | Kenya and DR Congo | Artemisinin-derivative drugs | Nine of the 24 drug samples analysed did not comply with the pharmacopoeial requirements; seven samples were under-dosed and two were slightly over-dosed. Dihydroartemisinin was the active ingredient in 57% of under-dosed samples.AE injections had the lowest drug content (77%). Two-thirds of the dry powder suspensions were either substandard or fake. | Atemnkeng *et al.* [34] |
| 2008 | Tanzania | SP and Sulphamethoxypyrazine/pyrimethamine | Poor-quality anti-malarials were common; 12.2% all samples were substandard, and the figure was as high as 23.8% for quinine. | Kaur *et al.* [35] |
| 2008 | Ghana, Kenya, Nigeria, Tanzania, Uganda | Artemisinin and its derivatives | 35% all samples tested failed either or both thin-layer chromatography and dissolution tests, and were substandard.  35% drugs originating in Asia failed either or both thin-layer chromatography and dissolution tests, and were substandard. However, locallyproduced drugs fared worse, with nearly half found to be substandard. | Bate *et al.* [36] |
| 2008 | Burkina Faso | AQ, SP, (ART); QN, CQ, and artemether-lumefantrine | A survey found that 10.6% drugs procured from licensed sellers were substandard, and 90% purchased from unlicensed sellers were substandard | Tipke *et al.* [3] |
| 2009 | Nigeria | QN | 46% either lacking the active ingredients or containing suboptimal quantities of the active ingredients. | Onwujekwe *et al.* [37] |
| 2009 | Nigeria | SP | 39% did not meet US Pharmacopoeia specifications for amount of active ingredients. | Onwujekwe *et al.* [37] |
| 2009 | Tanzania | Metakelfin | Discovered in 40 pharmacies. Metakelfin (anti-malarial) lacked sufficient active ingredient. | WHO [8] |
| 2009 | Lao PDR | Oral ART | A wide variety of wrong active ingredients were found. 14.8% fakes contained detectable amounts of artemisinin. It could lead resistant development. | Sengaloundeth *et al.* [38] |
| 2012 | Amazon Basin | Artemisinin derivatives | 11.6% were found not to meet quality specifications. Most failures were reported during visual and physical inspection, 8.5% and most of these were due to expired medicines, 83.1%. | Petralanda*.* [39] |
| 2012 | Guyana | Artemisinin derivatives | 45 of 77 (58%) anti-malarial medicines sampled in Guyana of which 30  failed visual & physical inspection and 18 failed quality control tests. | Evans *et al.* [40] |
| 2012 | Suriname | Primaquine and artemisinin derivatives | All Artecom samples were found to lack a label claim for primaquine, thus failing visual and physical inspection. | Evans *et al.* [40] |
| 2012 | India | NA | 7% of a sample of anti-malarial, TB and antibiotic drugs tested in major Indian cities failed quality testing | Bate [41] |

Note:AQ - amodiaquine; SP - sulphadoxine-pyrimethamine; ART- artesunate; QN - quinine; PRO - proguanil; CQ - chloroquine; MQ - mefloquine.

**References to papers cited in Additional file 1:**

1. WHO: *The quality of antimalarials: a study in seven African countries*. World Health Organization, Geneva, Switzerland; 2003. Available at <http://whqlibdoc.who.int/hq/2003/WHO_EDM_PAR_2003.4.pdf>. Accessed on 10th June 2013.
2. Newton P, Proux S, Green M, Smithuis F, Rozendaal J, Prakongpan S, Chotivanich K, Mayxay M, Looareesuwan S, Farrar J, Nosten F, White NJ: **Fake artesunate in Southeast Asia**. *Lancet* 2001, **357**:1948-1950.
3. Tipke M, Diallo S, Coulibaly B, Storzinger D, Hoppe-Tichy T, [Sie A](http://www.ncbi.nlm.nih.gov/pubmed?term=Sie A%5BAuthor%5D&cauthor=true&cauthor_uid=18505584), [Müller O](http://www.ncbi.nlm.nih.gov/pubmed?term=Müller O%5BAuthor%5D&cauthor=true&cauthor_uid=18505584): **Substandard anti-malarial drugs in Burkina Faso**. *Malar J* 2008, **7**:95

DOI: 10.1186/1475-2875-7-95.

1. Dondorp AM, Newton PN, Mayxay M, Van Damme W, Smithuis FM, Yeung S, Petit A, Lynam AJ, Johnson A, Hien TT, McGready R, Farrar JJ, Looareesuwan S, Day NP, Green MD, White NJ: **Fake antimalarials in Southeast Asia are a major impediment to malaria control: multinational cross-sectional survey on the prevalence of fake antimalarials**. *Trop Med Int Health* 2004, **12**:1241-1246.
2. Hall KA, Newton PN, Green MD, De Veij M, Vandenabeele P, Pizzanelli D, Mayxay M, Dondorp A, Fernandez FM: **Characterization of counterfeit artesunate antimalarial tablets from southeast Asia**. *Am J Trop Med Hyg* 2006, **75**:804-811.
3. Ambroise-Thomas P: **The tragedy caused by fake antimalarial drugs**. *Mediterr J Hematol Infect Dis* 2012, **4**:e2012027. DOI 10.4084/MJHID.2012.027.
4. Rozendaal J: **Fake antimalaria drugs in Cambodia**. *Lancet* 2001, **357**:890.
5. WHO: **Medicines: spurious/falsely-labelled/ falsified/counterfeit (SFFC) medicines**. Fact sheet N°275; 2012. <http://www.who.int/mediacentre/factsheets/fs275/en/>. Accessed on 08^th^ June 2013.
6. WHO: **Executive Board 124th session provisional agenda item 4.11**. Counterfeit medical products. EB124/14; 2008. <http://www.who.int/gb/ebwha/pdf_files/EB124/B124_14-en.pdf>.
7. Newton PN, Green MD, Fernández FM: **Impact of poor-quality medicines in the ‘developing’ world**. *Trends Pharmacol Sci* 2010, **31**:99-101.
8. Sesay MM: **Fake drugs - a new threat of health care delivery**. *Africa Health* June/July 1988, 13-15.
9. ten Ham M: **Counterfeit drugs: implications for health**. *Adverse Drug React Toxicol Rev* 1992, **11**:59-65.
10. WHO: *La qualité des médicaments sur le marché pharmaceutique africain. Etude analytique dans trois pays. Cameroun, Madagascar, Tchad.* World Health Organization, Geneva, Switzerland. WHO/DAP/95.3; 1995.(In French).
11. Petralanda I: **Quality of antimalarial drugs and resistance to *Plasmodium vivax* in Amazonian region**. *Lancet* 1995, **345**:1433.
12. Abdi YA, Rimoy G, Ericsson O, Alm C, Massele AY: **Quality of chloroquine preparations marketed in Dar es Salaam, Tanzania**. *Lancet* 1995, **346**:1161.
13. Kron MA: **Substandard primaquine phosphate for US Peace Corps personnel**. *Lancet* 1996, **348:**1453-1454.
14. Shakoor O, Taylor RB, Behrens RH: **Assessment of the incidence of substandard drugs in developing countries.** *Trop Med Int Health* 1997, **2:**839-845.
15. Ogwal-Okeng JW, Okello DO, Odyek O: **Quality of oral and parenteral chloroquine in Kampala.** *East Afr Med J* 1998, **75**:692-694.
16. Reidenberg MM, Conner BA: **Counterfeit and substandard drugs**. *Clin Pharmacol Ther* 2001, **69**:189-193.
17. Risha PG, Shewiyo D, Msami A, Masuki G, Vergote G, Vervaet C, Remon JP: ***In vitro* evaluation of the quality of essential drugs on the Tanzanian market**. *Trop Med Int Health* 2002, **7**:701-707.
18. Kapp C: **Counterfeit drug problem “underestimated”, says conference**. *Lancet* 2002, **360**:1080.
19. Wolff JC, Thomson LA, Eckers C: **Identification of the ‘wrong’ active pharmaceutical ingredient in a counterfeit Halfan drug product using accurate mass electrospray ionisation mass spectrometry, accurate mass tandem mass spectrometry and liquid chromatography/mass spectrometry**. *Rapid Comm Mass Spectrom* 2003, **17:**215-221.
20. Ogwal-Okeng JW, Owino E, Obua C: **Chloroquine in the Ugandan market fails quality test: a pharmacovigilance study**. *Afr Health Sci* 2003, **3:**2-6.
21. Minzi OMS, Moshi MJ, Hipolite D, Massele AY, Tomson G, Ericsson O, Gustafsson LL: **Evaluation of the quality of amodiaquine and sulphadoxine/pyrimethamine tablets sold by private wholesale pharmacies in Dar Es Salaam Tanzania***. J Clin Pharm Ther* 2003, **28**:117-122.
22. Basco LK: **Molecular epidemiology of malaria in Cameroon. XIX. Quality of antimalarial drugs used for self-medication**. *Am J Trop Med Hyg* 2004, **70**:245-250.
23. Kayumba PC, Risha PG, Shewiyo D, Msami A, Masuki G,  Ameye D, Vergote G, Ntawukuliryayo JD, Remon JP, Vervaet C: **The quality of essential antimicrobial and antimalarial drugs marketed in Rwanda and Tanzania: influence of tropical storage conditions on in vitro dissolution**. *J Clin Pharm Ther* 2004, **29**:331-338.
24. Smine A, Phanouvong S, Chanthap L, Tsuyuoka R, Nivana N, Blum N: Antimalarial Drug Quality in Mekong Countries. 2003. http://www.uspdqi.org/pubs/other/AntimalarialPoster.pdf. Accessed on 10 June 2013.
25. Amin AA, Kokwaro GO: **Antimalarial drug quality in Africa**. *J Clin Pharm Ther* 2007, **32**:429-440. DOI: 10.1111/j.1365-2710.2007.00847.x.
26. Legris C: **La détection des médicaments contrefaits par investigation de leur authenticité. Etude pilote sur le marché pharmaceutique illicite de Côte d’Ivoire.** These pour le diplome d’Etat de docteur en pharmacie, Faculté de Pharmacie Nancy, 1995. (in French) Available at: http://www.remed.org/html/theses.html. Accessed on 16^th^ May 2013.
27. Lon CT, Tsuyuoka R, Phanouvong S, Nivanna N, Socheat D, Sokhan C, Blum N, Christophel EM, Smine A: **Counterfeit and substandard antimalarial drugs in Cambodia**. *Trans R Soc Trop Med Hyg* 2006, **100**:1019-1024.
28. Vijaykadga S, Cholpol S, Sitthimongkol S, Pawaphutanan A, Pinyoratanachot A, Rojanawatsirivet C, Kovithvattanapong R, Thimasarn K: **Strengthening of national capacity in implementation of antimalarial drug quality assurance in Thailand**. *Southeast Asian J Trop Med Pub Health* 2006, **37**:5-10.
29. McGivering J: **Tracking the fake malaria drug threat**. BBC News; 2007. Available at: http://news.bbc. co.uk/1/hi/world/asia-pacific/6692431.stm. Accessed on 09^th^ June 2013.
30. Gaudiano MC, Di Maggio A, Cocchieri E, Antoniella E, Bertocchi P, Alimonti S, Valvo L: **Medicines informal market in Congo, Burundi and Angola: counterfeit and sub-standard anti-malarials.** Malar J 2007, **6:**22.
31. Atemnkeng MA, De Cock K, Plaizier-Vercammen J: **Quality control of active ingredients in artemisinin-derivative antimalarials within Kenya and DR Congo**. *Trop Med Int Health* 2007, **12**:68-74.
32. Kaur H, Goodman C, Thompson E, Thompson K-A, Masanja I, Kachur SP, Abdulla S: **A nationwide survey of the quality of antimalarials in retail outlets in Tanzania.** *PLoS ONE* 2008, **3**:e3403.
33. Bate R, Coticelli P, Tren R, Attaran A: **Antimalarial drug quality in the most severely malarious parts of Africa – A six country study**. *PLoS ONE* 2008, **3**:e2132.
34. Onwujekwe O, Kaur H, Dike N, Shu E, Uzochukwu B, Hanson K, Okoye V, Okonkwo P: **Quality of anti-malarial drugs provided by public and private healthcare providers in south-east Nigeria.** Malar J 2009, **8:**22.
35. Sengaloundeth S, Green MD, Fernandez FM, Manolin O, Phommavong K, Insixiengmay V, Hampton CY, Nyadong L, Mildenhall DC, Hostetler D, Khounsaknalath L, Vongsack L, Phompida S, Vanisaveth V, Syhakhang L, Newton PN: **A stratified random survey of the proportion of poor quality oral artesunate sold at medicine outlets in the Lao PDR-implications for therapeutic failure and drug resistance**. *Malar J* 2009, **8**:172.
36. Petralanda I: **Quality of antimalarial drugs and resistance to *Plasmodium vivax* in Amazonian region.** *Lancet* 1995, **345**:1433.
37. Evans L 3rd, Coignez V, Barojas A, Bempong D, Bradby S, [Dijiba Y](http://www.ncbi.nlm.nih.gov/pubmed?term=Dijiba Y%5BAuthor%5D&cauthor=true&cauthor_uid=22704709), [James M](http://www.ncbi.nlm.nih.gov/pubmed?term=James M%5BAuthor%5D&cauthor=true&cauthor_uid=22704709), [Bretas G](http://www.ncbi.nlm.nih.gov/pubmed?term=Bretas G%5BAuthor%5D&cauthor=true&cauthor_uid=22704709), [Adhin M](http://www.ncbi.nlm.nih.gov/pubmed?term=Adhin M%5BAuthor%5D&cauthor=true&cauthor_uid=22704709), [Ceron N](http://www.ncbi.nlm.nih.gov/pubmed?term=Ceron N%5BAuthor%5D&cauthor=true&cauthor_uid=22704709), [Hinds-Semple A](http://www.ncbi.nlm.nih.gov/pubmed?term=Hinds-Semple A%5BAuthor%5D&cauthor=true&cauthor_uid=22704709), [Chibwe K](http://www.ncbi.nlm.nih.gov/pubmed?term=Chibwe K%5BAuthor%5D&cauthor=true&cauthor_uid=22704709), [Lukulay P](http://www.ncbi.nlm.nih.gov/pubmed?term=Lukulay P%5BAuthor%5D&cauthor=true&cauthor_uid=22704709), [Pribluda V](http://www.ncbi.nlm.nih.gov/pubmed?term=Pribluda V%5BAuthor%5D&cauthor=true&cauthor_uid=22704709): **Quality of anti-malarials collected in the private and informal sectors in Guyana and Suriname**. *Malar J* 2012, **11**:203.
38. Bate R: **Phake: the deadly world of falsified and substandard pharmaceuticals.** Washington, DC: The American Enterprise Institute Press, 2012.
39. Stevens P, Mydin HH: **EMHN briefing no.1: Fake Medicines in Asia;** 2013. http://www.emhn.org/articles/emhn-briefing-no1-fake-medicines-asia. Accessed on 30th May 2013.
